# Experiences With Prenatal Care Delivery Reported by Black Patients With Low Income and by Health Care Workers in the US: A Qualitative Study

**DOI:** 10.1001/jamanetworkopen.2022.38161

**Published:** 2022-10-24

**Authors:** Alex Friedman Peahl, Michelle H. Moniz, Michele Heisler, Aalap Doshi, Gwendolyn Daniels, Martina Caldwell, Vanessa K. Dalton, Ana De Roo, Mary Byrnes

**Affiliations:** 1 Department of Obstetrics and Gynecology, University of Michigan, Ann Arbor; 2 Institute for Healthcare Policy and Innovation, University of Michigan, Ann Arbor; 3 Program on Women’s Healthcare Effectiveness Research, University of Michigan, Ann Arbor; 4 Department of Internal Medicine, University of Michigan, Ann Arbor; 5 Michigan Institute for Clinical and Health Research, University of Michigan, Ann Arbor; 6 Institute for Population Health, Detroit, Michigan; 7 Department of Emergency Medicine, Henry Ford Health System, Detroit, Michigan; 8 Department of Surgery, University of Michigan, Ann Arbor; 9 Center for Healthcare Outcomes and Policy, University of Michigan, Ann Arbor

## Abstract

**Importance:**

Black pregnant people with low income face inequities in health care access and outcomes in the US, yet their voices have been largely absent from redesigning prenatal care.

**Objective:**

To examine patients’ and health care workers’ experiences with prenatal care delivery in a largely low-income Black population to inform care innovations to improve care coordination, access, quality, and outcomes.

**Design, Setting, and Participants:**

For this qualitative study, human-centered design–informed interviews were conducted at prenatal care clinics with 19 low-income Black patients who were currently pregnant or up to 1 year post partum and 19 health care workers (eg, physicians, nurses, and community health workers) in Detroit, Michigan, between October 14, 2019, and February 7, 2020. Questions focused on 2 human-centered design phases: observation (understanding problems from the end user’s perspective) and ideation (generating novel potential solutions). Questions targeted participants’ experiences with the 3 goals of prenatal care: medical care, anticipatory guidance, and social support. An eclectic analytic strategy, including inductive thematic analysis and matrix coding, was used to identify promising strategies for prenatal care redesign.

**Main Outcomes and Measures:**

Preferences for prenatal care redesign.

**Results:**

Nineteen Black patients (mean [SD] age, 28.4 [5.9] years; 19 [100%] female; and 17 [89.5%] with public insurance) and 17 of 19 health care workers (mean [SD] age, 47.9 [15.7] years; 15 female [88.2%]; and 13 [76.5%] Black) completed the surveys. A range of health care workers were included (eg, physicians, doulas, and social workers). Although all affirmed the 3 prenatal care goals, participants reported failures and potential solutions for each area of prenatal care delivery. Themes also emerged in 2 cross-cutting areas: practitioners and care infrastructure. Participants reported that, ideally, care structure would enable strong ongoing relationships between patients and practitioners. Practitioners would coordinate all prenatal services, not just medical care. Finally, care would be tailored to individual patients by using care navigators, flexible models, and colocation of services to reduce barriers.

**Conclusions and Relevance:**

In this qualitative study of low-income, Black pregnant people in Detroit, Michigan, and the health care workers who care for them, prenatal care delivery failed to meet many patients’ needs. Participants reported that an ideal care delivery model would include comprehensive, integrated services across the health care system, expanding beyond medical care to also include patients’ social needs and preferences.

## Key Points

**Question:**

How could prenatal care be redesigned to improve access, quality, and experience for Black pregnant people with low income who face significant inequities in prenatal care delivery and outcomes?

**Findings:**

In this qualitative study, patients and health care workers confirmed prenatal care delivery did not meet patients’ needs or preferences. Participants’ ideal prenatal care model would be anchored by a supportive practitioner in collaboration with a community-based team using integrated, flexible care delivery to meet patients’ diverse needs.

**Meaning:**

These findings suggest that prenatal care redesign can inspire new care models to address persistent inequities in prenatal care delivery and outcomes.

## Teaser

This qualitative study examines the prenatal care delivery experience of health care workers and patients in a low-income Black population.

## Introduction

Maternity care in the US is marred by significant health inequities, with Black and low-income people 2 to 5 times more likely to die in childbirth or experience severe maternal morbidity than their White counterparts.^1,2^ Prenatal care is an important upstream target to reduce maternal deaths and morbidity, yet prior work^3,4^ has demonstrated that Black people—particularly those with low socioeconomic status living in urban areas—face significant barriers to high-quality care, including lack of transportation, financial constraints, structural racism, and explicit discrimination. Existing prenatal care delivery structures that require frequent in-person contacts in clinical settings can exacerbate these barriers.^5,6,7,8,9,10^ Thus, prenatal care in its current form may actually create unjust barriers for those who stand to benefit the most from receiving this important health service.

Although Black and low-income individuals face significant care inequities, their voices have been largely absent from prenatal care delivery research. Thus, clinicians and health care leaders lack important information to redesign prenatal care in line with these populations’ views. Human-centered design (HCD), a method for redesigning processes from the end user’s perspective, has successfully generated new prenatal care models tailored for both patients and health care workers (HCWs).^11,12,13,14^ Human-centered design could be an effective strategy for reimagining prenatal care for the specific needs of low-income and Black patients^15^; however, to date, HCD work has been conducted with largely White, highly educated, high-income populations in well-resourced academic care settings.^11^

This study was conducted in Detroit, Michigan, which has a long history of health disparities and racial injustice, with significantly higher rates of maternal and infant morbidity and mortality than the US average.^16,17,18^ Our objective was to explore patients’ and HCWs’ experiences with current prenatal care delivery using HCD to identify methods of improving care for low-income, Black pregnant people. This report is part of a larger project to optimize prenatal care for pregnant people in the safety net by improving prenatal care access, patient experience, and clinical outcomes.

## Methods

In this qualitative study, we used HCD-informed interviews to center patients’ and HCWs’ voices in redesigning prenatal care. We completed the first 2 HCD phases: observation (understanding the problem from the end user’s perspective) and ideation (generating novel potential solutions)^19^ (eTable 1 in the Supplement). We addressed all 3 goals of prenatal care: medical care (routine prenatal visits to screen for and manage comorbidities and pregnancy complications), anticipatory guidance (education about pregnancy, childbirth, the postpartum period, and parenting), and social support (nonmedical factors that impact patients’ ability to access and engage with the health system, including material resources [eg, housing and transportation] and social support [eg, relationships, community, and emotional support]).^8^ eTable 2 in the Supplement details the interview domains. This study was deemed not regulated by the University of Michigan Institutional Review Board as a quality improvement activity to evaluate existing prenatal care services and develop new programs. Written informed consent was obtained from all participants. This qualitative study followed the Consolidated Criteria for Reporting Qualitative Research (COREQ) reporting guideline.

We offered semistructured interviews with 19 low-income, Black, pregnant and postpartum patients and the 19 HCWs who care for them, including obstetrician-gynecologists, midwives, nurses, nutritionists, community health workers, and doulas. Interviews were conducted from October 14, 2019, to February 7, 2020. We designed an HCD-informed interview script in collaboration with experts in HCD, qualitative methodology, and prenatal care. For the observation phase, we used journey mapping, in which participants describe their (or their clients’) prenatal care experience from pregnancy discovery through delivery.^19^ Previous studies demonstrated that this approach can elucidate opportunities during pregnancy in which more, less, or different care would have been preferred.^13,20,21^ For ideation, we asked participants to imagine their ideal prenatal care experience.

We developed a survey for key participant demographic characteristics (eg, race and ethnicity), all of which were self-reported by the participant. Patient-specific fields included pregnancy and prenatal care information (eg, parity and practitioner). The HCW information included professional characteristics (eg, years in practice). All items were derived from prior surveys conducted in this population.^22,23^ We reviewed preliminary materials with 2 Detroit community HCWs to ensure questions were clear, acceptable, and comprehensive.

With our community partners, we identified 2 clinics that provide prenatal care to predominantly Black, low-income pregnant people in Detroit. We conducted 2 exploratory 90-minute focus groups (n = 14) to gain preliminary insights on interview domains and ensure the acceptability of questions. Participants received a $40 gift card as compensation. After focus groups, additional prompts were added to the interview guide. Focus group data were not included in the final analysis.

For interviews, we used snowball sampling—a methodologic approach in which existing participants identify future participants to recruit as information-rich key informants.^24^ Before the interviews, participants were screened by telephone for eligibility: age older than 18 years, English speaking, and a confirmed pregnancy at any gestational age or up to 1 year post partum. All HCWs (ie, anyone who provides services for pregnant people) at the study sites were eligible.^25^ We conducted 38 interviews to reach information power—the point at which sufficient qualitative data have been collected to rigorously answer the research question.^26^ On the basis of prior literature,^5,27^ the nature of our research question, the specificity of the recruited sample, and the planned depth of interviews, we estimated that between 15 and 20 interviews per group (patients and HCWs) would be required to answer the research question. Interviews were held at local clinics and timed with prenatal care or HCW breaks. Participants completed the survey before their 60- to 90-minute interview and received a $40 gift card as compensation.

Interviews were recorded, transcribed verbatim, reviewed for accuracy, and managed with qualitative coding software (MaxQDA, version 2020.0.0; VERBI GmbH). We used an eclectic analytic strategy that included inductive thematic and matrix analysis^28^: 2 authors (A.F.P. and M.B.) initially reviewed the data and open-coded 3 transcripts for patterns, concepts, and key ideas. Next, we built, tested, and iteratively refined the codebook based on initial categories. Three authors (A.F.P, M.B., and A.D.R.) independently applied main codes and subcodes to transcripts. Finally, we compared coding decisions and refined our codebook. We then built a matrix of the 3 prenatal care goals and 2 HCD concepts: (1) observation (“How is prenatal care failing to meet those goals?”) and (2) ideation (“How would prenatal care be delivered in an ideal setting?”). Representative quotes were combined in tables to identify emerging themes and findings. In February 2021, we completed 4 member-checking interviews (2 patients and 2 HCWs) to confirm that findings accurately represented participants’ experiences. No substantial changes were recommended.

## Results

Nineteen Black patients (mean [SD] age, 28.4 [5.9] years; 19 [100%] female) and 17 of the 19 HCWs (mean [SD] age, 47.9 [15.7] years; 15 female [88.2%]; and 13 [76.5%] Black, 1 [5.9%] Latinx, 3 [17.6%] White, and 1 [5.9%] of ≥2 races) completed the surveys. Most patients had public insurance (17 of 19 [89.5%]), were multiparous (17 of 19 [89.5%]), and saw an obstetrician-gynecologist during pregnancy (17 of 19 [89.5%]). Most HCWs cared for patients with public insurance (13 of 17 [76.5%]) (Table 1 and Table 2).

**Table 1.** Characteristics of 19 Participating Patients

Characteristic	Patients, No. (%)
Age, mean (SD), y	28.4 (5.9)
Race and ethnicity	
Black or African American	19 (100)
White	0
Latinx	1 (5.0)
Insurance	
Public (Medicaid)	17 (89.5)
Commercial	2 (10.5)
Educational level	
Some high school, no diploma	1 (5.3)
High school graduate, diploma or equivalent	8 (42.1)
Some college, no degree	6 (31.6)
Trade, technical, or vocational school	2 (10.5)
Associate’s degree	1 (5.3)
Bachelor’s degree	1 (5.3)
Advanced degree (master’s, professional, or doctorate)	0
Employment	
Employed for wages	3 (15.8)
Self-employed	1 (5.3)
Out of work	8 (42.1)
Unable to work	6 (31.6)
Homemaker	0
Student	1 (5.3)
Military	0
Retired	0
Relationship status	
Single	8 (42.1)
In a relationship	8 (42.1)
Married	2 (10.5)
Separated or divorced	1 (5.3)
Gravidity, median (IQR)	4 (2-5)
Parity, median (IQR)	2 (1-3)
Nulliparous patients	2 (10.5)
Chronic medical conditions	
Diabetes	1 (5.3)
Hypertension	3 (15.8)
Asthma	3 (15.8)
Depression	3 (15.8)
Prenatal care practitioner	
Obstetrician-gynecologist	17 (89.5)
Midwife	1 (5.3)
Both midwife and obstetrician gynecologist	1 (5.3)
Did you participate in group prenatal care?	
Yes	3 (15.8)
No	16 (84.2)
How satisfied were you with your prenatal care?	
Very satisfied	9 (47.4)
Satisfied	9 (47.4)
Dissatisfied	1 (5.3)
Very dissatisfied	0
How often do you have trouble understanding written materials about pregnancy?	
Sometimes	6 (31.6)
Never	13 (68.4)

**Table 2.** Characteristics of 17 Participating Health Care Workers (HCWs)

Characteristic	HCWs, No. (%)
Age, mean (SD), y	47.9 (15.7)
Sex	
Female	15 (88.2)
Male	2 (11.8)
Race and ethnicity	
Black or African American	13 (76.5)
White	3 (17.6)
Latinx	1 (5.9)
≥2 Races	1 (5.9)
Clinical position	
Obstetrician gynecologist	2 (11.8)
Emergency medicine physician	1 (5.9)
Midwife	1 (5.9)
Nurse	1 (5.9)
Community health worker	3 (17.6)
Doula or childbirth educator	3 (17.6)
Social worker	1 (5.9)
Nutritionist	3 (17.6)
Women, infants, and children customer service representative	1 (5.9)
Breastfeeding specialist	1 (5.9)
Lactation consultant	1 (5.9)
Dental hygienist	1 (5.9)
Duration in practice, y	
<1	1 (5.9)
1-5	8 (47.1)
5-10	2 (11.8)
>10	6 (35.3)
Main client insurance type	
Public (Medicaid)	13 (76.5)
Commercial	4 (23.5)
Satisfaction with current prenatal care model	
Very satisfied	1 (5.9)
Satisfied	9 (52.9)
Dissatisfied	6 (35.3)
Very dissatisfied	1 (5.9)

Interviews revealed important insights about the failures of current prenatal care delivery and potential solutions. Participants also identified 2 cross-cutting care areas for intervention: practitioners and prenatal care infrastructure (Table 3). We explore the following 5 aspects of prenatal care in the context of prenatal care goals, failures, and ideal care: medical care, anticipatory guidance, social support, practitioners, and care infrastructure.

**Table 3.** Failures and Ideal Future of Prenatal Care to Improve the Pregnancy Experience and Outcomes as Identified by Participants

Care domain	Failures of prenatal care	Ideal future of prenatal care
Medical care	Prenatal appointments often do not give patients clear medical benefit or reassurance	Patients should enter pregnancy healthy
	Prenatal visits are low value to many patients	Intensity of medical care in pregnancy should be based on risk factors
		Mental health should be integrated in pregnancy care
Anticipatory guidance	Inadequate reliable, easily accessible information	Pregnancy information should be comprehensive, clear, and integrated into prenatal care for all pregnancy stages
	Health care workers lack time and educational resources to share with patients	Patients should have safe spaces to ask questions and gain information
	Patients are not comfortable asking questions	
	Online resources and friends and family are readily available but unreliable	
Psychosocial support		
Material needs	Screening for resource needs is not sufficient	Prenatal care should support patients in meeting their basic needs, including housing, nutrition, and safety
	Accessing resources is complex and requires significant assistance	Prenatal care should support patients in obtaining health care coverage
	Available resources are insufficient	
Social support	Patients desire greater partner support (eg, father of the child or significant other)	Patients should have a supportive community to help them navigate pregnancy
	Current prenatal care structure does not integrate psychosocial support	Social support should be integrated into prenatal care
Maternity care professionals	Short appointments, seeing multiple maternity care professionals in pregnancy, and administrative burden preclude strong relationships between patients and maternity care professionals	Desire for meaningful relationships with maternity care professionals
	Maternity care professionals do not address patients’ nonmedical needs	Need for maternity care professionals to genuinely care about the patient
	Short appointments, seeing multiple maternity care professionals in pregnancy, and administrative burden preclude strong relationships between patients and maternity care professionals	Hope for the maternity care professional to coordinate medical and psychosocial aspects of prenatal care
Care infrastructure	Medical care, anticipatory guidance, and psychosocial support are poorly integrated	Care structure should be tailored to patients’ needs and preferences
	Patients struggle to receive care and balance other obligations	Prenatal care should be modified to decrease barriers
	Prenatal care is one size fits all and is not tailored for individuals	Additional care team members (eg, community health workers and doulas)
		Flexible care models
		Colocation of services

### Defining Goals of Prenatal Care

Patients and HCWs affirmed the 3 goals of prenatal care identified by the American College of Obstetricians and Gynecologists (Box; eTable 3 in the Supplement). Medical care goals included optimizing the pregnant patient’s and infant’s health and providing reassurance that the pregnancy was healthy. Anticipatory guidance was deemed important for preparing patients for pregnancy, birth, and parenting. Patients and HCWs believed all patients, regardless of educational level or parity, could benefit from information in pregnancy, although patients with less experience may benefit most. Finally, participants identified material and social support as crucial for facilitating prenatal care engagement.

Box. **Goals of Prenatal Care Outlined by the American College of Obstetricians and Gynecologists and Confirmed by Participants**

**Medical Care**

Optimize health
Provide reassurance

**Anticipatory Guidance**

Provide education on pregnancy, birth, the postpartum period, and parenting

**Psychosocial Support**

Identify material needs and provide resources
Provide social support

### Failures of Current Prenatal Care Delivery

Patients and HCWs reported that prenatal care often failed to address the 3 prenatal care goals (Table 3; eTable 4 in the Supplement). For medical care, participants believed routine prenatal visits often did not provide meaningful health benefits.

One client told me ‘I was pregnant before, I don’t have to go every time. And they don’t be doing anything anyway’…. she felt that her experience wasn’t worth it. (HCW 8)

Many patients similarly did not feel reassured by routine prenatal visits that simply informed them that the pregnancy was “getting further and further” (patient 11, multiparous, 23 weeks’ gestation). Therefore, patients and HCWs reported that prenatal visits were low value for some patients, particularly those who faced significant barriers to care, such as inadequate transportation. Of interest, the same prenatal care service had different meanings for patients relative to their social needs: whereas 1 patient reported reassurance from hearing the fetal heartbeat, another who faced homelessness found this less valuable:

She’s [the doctor] just going to check the heartbeat and it’s going to be like 10 minutes. No, I’m not going to waste my gas. Back then, I was surviving. (patient 6, multiparous, post partum)

For some pregnant patients, particularly those who are “surviving,” it may not be worth expending resources simply to be told they are getting “further and further.”

Patients and HCWs also identified gaps in anticipatory guidance, including lack of high-quality, easily accessible resources for routine pregnancy information. Often, HCWs lacked time to provide anticipatory guidance, whereas many patients were hesitant to ask their care team questions:

When you ask a lot of questions and then it looks like you don’t know what you’re talking about…you don’t want to look like you don’t know about your own body. (patient 24, multiparous, 20 weeks’ gestation)

As a result, patients often sought information online or through friends and family. Although these resources were more accessible, HCWs believed these resources frequently conveyed incorrect information. Participants were thus sometimes trapped in a cycle of misinformation, without tools to systematically address educational needs.

Patients and HCWs identified several gaps in support for material needs, including inconsistent screening, insufficient resources, and a complex process for accessing available services. One reflected, “It will take you all day to find an actual good resource” (patient 12, multiparous, 20 weeks’ gestation).

Social support was also often described as inadequate. Many patients reflected on the limited support they received from their partners: “My friend is more supportive than my boyfriend” (patient 10, multiparous, 26 weeks’ gestation). Similarly, HCWs recognized that current prenatal care delivery did not routinely provide patients with needed support in a variety of forms—emotional and physical support as well as comfort—that could be better addressed through prenatal care.

Patients and HCWs identified problems with current patient-professional relationships. Short appointments with different practitioners, particularly in academic clinics, frequently made it difficult to establish meaningful relationships: “I think a lot of times people just don’t feel like they know who they are talking to...” (HCW 5). Having to share their personal information repeatedly with new people made some patients feel their practitioner did not care about them. Likewise, HCWs described barriers to developing meaningful relationships with patients:

They need to strip the administrative blah, blah, blah, out of prenatal care…because [if] you’re not under the gun to turn over patients every few minutes…you could answer more questions. (HCW 17)

Finally, participants believed many practitioners do not routinely and systematically address nonmedical needs despite the tripartite goals of prenatal care.

They [providers] may not even know kind of what the family or the mother may need or want… I don’t think it’s enough just to have the provider come in and do their thing. (HCW 5)

This HCW acknowledged that practitioners may not be aware of or trained to manage nonmedical needs, but there “needs to be more support” in place.

Patients and HCWs identified structural issues with prenatal care delivery. Decentralized resources made fulfilling medical and nonmedical needs challenging. In addition, many patients reported difficulty balancing other obligations, such as work and childcare, with prenatal services.

Finally, participants reflected that prenatal care failed to account for patients’ individual needs. As one HCW described, highlighting the importance of individualizing care:

They [providers] shouldn’t have to always be just a clinic mode, and this is exactly how we do it, this is our cookie cutter. (HCW 11)

### Ideal Prenatal Care Delivery

Although patients and HCWs identified flaws in the prenatal care system, they shared a vision for redesigning it (Table 3; eTable 5 in the Supplement). Specifically for medical care, HCWs hoped patients could enter pregnancy healthy, without “any chronic illnesses that goes along with it” (HCW 4). They also envisioned a system of risk-based medical care adjusted to patients’ needs. One HCW suggested, “if it’s not high risk, it shouldn’t be treated as high risk” (HCW 7), reflecting a plan for escalation and de-escalation of care. Finally, participants recognized the importance of having mental health resources “automatically” available for all patients (HCW 2).

Patients and HCWs identified 3 key solutions for improving anticipatory guidance in pregnancy. Many suggested organized curricula that systematically covers information in a “trimester-specific” fashion (HCW 18). Participants envisioned comprehensive topics, including mental health and parenting, emphasizing that materials should be clear, understandable, and in a “user-friendly format” (HCW 3). Last, many described a critical need for a safe space to ask questions:

You’ve got a suicide hotline, why can’t you have a pregnancy hotline… Some people aren’t able to say what it is that they need, what it is that they want and answers that they want to get. (patient 3, multiparous, post partum)

This perspective suggests that patients may fear being judged for asking questions in routine clinical settings, necessitating an anonymous place to seek information.

Patients and HCWs agreed that an ideal pregnancy would include basic material needs (eg, food, housing, and a safe environment) and reliable health care coverage so patients would “not have to worry about the insurance” (HCW 4). Many HCWs thought ideal social support in pregnancy would involve a community approach. One shared,

I think the perfect pregnancy experience would be a time for excitement, a time for support—like rallying the whole village around, I guess. (HCW 5)

Health care workers described this support built into routine care through models such as group prenatal care, in addition to the informal support many already provided.

Patients and HCWs believed the ideal practitioner would develop strong relationships with patients. One patient described this relationship as “…her [the doctor] just being supportive, asking plenty of questions… not being rushed” (patient 5, multiparous, post partum). Patients believed the practitioner should genuinely care about them, “because if the doctor don’t care about your body, how are you supposed to know what’s going on?” (patient 12, multiparous, 20 weeks’ gestation). To this patient, the physician’s medical knowledge was not a replacement for genuinely caring about the patient and her “body.”

In addition, in patients’ and HCWs’ ideal model, practitioners would be the center of all aspects of prenatal care:

They [providers] have to be all things…Actually addressing all concerns...And if it’s something they cannot address, they need to make sure that they’re putting their patient with the appropriate person to be able to address it. (HCW 7)

Building on this idea, participants stated that an ideal system would include better coordination between the primary practitioner and other team members, including doulas or subspecialists for high-risk pregnancies.

Finally, 2 themes emerged around ideal prenatal care delivery structure: (1) tailored care and (2) modifications to eliminate barriers. Participants emphasized the importance of tailoring services to patients’ needs and preferences, identified from the beginning of pregnancy:

Care should always be personalized…I think that moms would feel like they are more involved in their care and maybe would be more likely to come to appointments if they feel like, oh, I have set out this path for myself so I will show up. (HCW 2)

Patients and HCWs suggested 3 modifications to care delivery to overcome barriers: care navigators, flexible prenatal care models, and colocation of services. Participants envisioned a robust network of care navigators, such as community HCWs or doulas, who could help patients accomplish health care tasks and feel supported. They also identified flexible prenatal care models (eg, telemedicine, group care, and community-based clinics) and expanded hours, availability of childcare services, and colocation of medical and nonmedical services as avenues for reducing access barriers. One HCW’s vision of ideal prenatal care demonstrated this best:

It’s already in my head: a housing program that you make sure that they are stabilized…then they’re attached to other resources like, a one-stop shop. The Ob/Gyn [obstetrician-gynecologist] and the MIHP [Maternal Infant Health Program], which includes the social worker, registered dietitian, then they would need a resource center to make sure that they’re going to be cared for…that person is going to be able to have a chance. (HCW 15)

In summary, participants’ vision of the ideal prenatal care structure was an integrated, holistic approach to pregnancy care—designed for patients by patients, with adequate support to meet all needs.

## Discussion

In this HCD-informed qualitative study, low-income Black patients and HCWs reported that the goals of prenatal care are not consistently met through the existing prenatal care delivery system, particularly for patients facing significant adverse social and structural determinants of health. The current medicalized care structure further precludes realization of these goals by perpetuating a standard, siloed approach to care delivery. Participants’ ideal prenatal care model, in contrast, was centered on an engaged, supportive practitioner anchored in a community-based team, with an integrated, flexible structure of care delivery capable of meeting patients’ diverse needs during and beyond pregnancy.

Recent efforts, including the Centers for Disease Control and Prevention’s Hear Her campaign, have elevated patients’ voices as a critical part of recognizing pregnancy complications and preventing maternal morbidity and mortality.^29^ Our HCD work similarly incorporates patients’ voices into the development of a new prenatal care system to facilitate safety, support, and satisfaction during and after pregnancy.

Other studies of prenatal care emphasize the need for more flexible care models, with telemedicine contacts, increased education, and improved social support.^11,22,30^ Our study expands these findings to a largely low-income Black population that has faced significant maternity care inequities in both access and outcomes.^16,17,18^

Many barriers preclude realization of the ideal prenatal care model. We lack standard screening tools to define people’s diverse medical, anticipatory guidance, and social needs and desire for assistance. Even once needs are identified, we lack guidance on how to partner with patients to develop tailored care plans. Although participants identified the practitioner as the ideal hub for comprehensive prenatal care planning, these individuals often lack training in nonmedical domains. Team-based care approaches, such as those used in pediatrics and internal medicine, can facilitate comprehensive patient care while preventing burnout.^31,32,33,34^ Uptake of care models that improve accessibility, including telemedicine and group care, are limited by payer coverage and logistical support, such as broadband internet, clinic space, and access to high-quality home monitoring devices, such as blood pressure cuffs.^9,35,36^ Policy changes, including payment reform, maintenance of telemedicine policies enacted during the COVID-19 public health crisis, and greater investment in social and structural determinants of health, are needed to ensure people who may benefit most from flexible models of care can access them.

For the next steps of the HCD process, we will select the most promising ideas generated through this initial work to develop and study interventions with key community stakeholders. Through rapid prototyping, user feedback, iteration, and implementation, we will develop and refine interventions designed for pregnant people and those who care for them.

Many of the gaps in care delivery highlighted in this study, including the need for individualized care plans and better integration of social and structural determinants of health, became rapidly apparent during the COVID-19 public health crisis, when social distancing and economic hardship exacerbated existing disparities in maternity care access and outcomes. A new national prenatal care recommendation, the Plan for Appropriate Tailored Healthcare (PATH) in pregnancy based on experience during the COVID-19 pandemic, emerging evidence, expert opinion, and patient input, seeks to improve prenatal care delivery using a more flexible, comprehensive approach.^37,38^ Recommendations include screening for medical, social, and structural determinants from the beginning of pregnancy; designing individualized prenatal care plans; and ensuring patients are connected with community and health system resources. New prenatal care delivery recommendations are an important step toward a more patient-centered approach to prenatal care—designed for patients with patients’ input.

### Limitations

Although our study provides important insights on prenatal care delivery, we acknowledge its limitations. First, our sample is limited to patients who were engaged in prenatal care, receiving care at safety net clinics, and seen in an urban setting; therefore, our findings may not generalize to those unable to access routine outpatient services, receiving prenatal care in other practice settings, or living in rural locations. Second, interviews were conducted by a White obstetrician-gynecologist from a neighboring city; however, we took multiple steps to ensure participants’ comfort, including establishing rapport with local community leaders, beginning interviews with handoffs from trusted patients and HCWs whenever possible, and including research assistants from the community in interviews. The depth and intimacy of the voices captured in these interviews reflect the willingness of our participants to share their most personal experiences to help improve prenatal care delivery for future patients.

## Conclusions

In this qualitative study of low-income, Black pregnant people in Detroit, Michigan, and the HCWs who care for them, prenatal care delivery failed to meet the goals and preferences of marginalized populations. Future work is needed to translate the ideal prenatal care model described by participants into reality. We look forward to codesigning new, flexible, tailored prenatal care interventions that focus on whole patients in the context of their lives, preferences, and communities.

## Notes

Supplement. **eTable 1.** Key Elements of the Human-Centered Design Process **eTable 2.** Interview Script for HCD-Informed Interviews **eTable 3.** Goals of Prenatal Care as Identified by Participants **eTable 4.** Failures of Current Prenatal Care Delivery to Improve the Pregnancy Experience and Outcomes **eTable 5.** Ideal Future of Prenatal Care to Improve the Pregnancy Experience and Outcomes
