# Supplementary material for: Experiences With Prenatal Care Delivery Reported by Black Patients With Low Income and by Health Care Workers in the US: A Qualitative Study
Source: JAMA Netw Open. 2022 Oct 24;5(10):e2238161. doi: 10.1001/jamanetworkopen.2022.38161 (PMC9593232; doi:10.1001/jamanetworkopen.2022.38161)
Supplement: Supplement. — eTable 1. Key Elements of the Human-Centered Design Process eTable 2. Interview Script for HCD-Informed Interviews eTable 3. Goals of Prenatal Care as Identified by Participants eTable 4. Failures of Current Prenatal Care Delivery to Improve the Pregnancy Experience and Outcomes eTable 5. Ideal Future of Prenatal Care to Improve the Pregnancy Experience and Outcomes [file jamanetwopen-e2238161-s001.pdf]

Supplementary Online Content

Peahl AF, Moniz MH, Heisler M, et al. Experiences with prenatal care delivery reported by Black patients with low income and by health care workers in the US: a qualitative study. *JAMA Netw Open*. 2022;5(10):e2238161. doi:10.1001/jamanetworkopen.2022.38161

eTable 1. Key Elements of the Human-Centered Design Process

eTable 2. Interview Script for HCD-Informed Interviews

eTable 3. Goals of Prenatal Care as Identified by Participants

eTable 4. Failures of Current Prenatal Care Delivery to Improve the Pregnancy Experience and Outcomes

eTable 5. Ideal Future of Prenatal Care to Improve the Pregnancy Experience and Outcomes

This supplementary material has been provided by the authors to give readers additional information about their work.

© 2022 Peahl AF et al. *JAMA Network Open.*

eTable 1. Key Elements of the Human-Centered Design Process[1]

Human-Centered Design Step	Process	Prenatal Care Example
**Observation**	The design team gathers information on the problem from the users' perspective	*How do people giving and receiving prenatal care view it?*
**Ideation**	The design team generates multiple novel potential solutions	*What are creative solutions that address end users' needs?*
**Rapid prototyping**	The design team creates low-fidelity (simple, low-technology) models to be shared with users	*What could prenatal care solutions look like?*
**User feedback**	The design team seeks input from the end users to assess prototype success	*What do key stakeholders think of proposed solutions?*
**Iteration**	Recommendations are incorporated in new prototypes until consensus is reached	*How can we modify our initial thoughts to respond to users' feedback?*
**Implementation**	The ideas are trialed in real settings	*How does the idea actually work in practice?*

[1]IDEO.org. The Field Guide to Human-Centered Design. San Francisco, CA: IDEO.org; 2015

© 2022 Peahl AF et al. *JAMA Network Open.*

eTable 2. Interview script for HCD-informed interviews

Domain	Sample Question
Human-Centered Design	Journey Mapping (memorable moments, challenges, emotions in pregnancy)
Human-Centered Design	Imagine a future without any limitations and describe what you hope the pregnancy experience will be like for (your/your clients') children.
Medical Services	Tell me about your experience with regular outpatient prenatal care with your provider.
Anticipatory Guidance	Think about a time you needed more information in pregnancy; what did you do?
Psychosocial Support	What support did you receive in pregnancy? What support do you wish you had?

© 2022 Peahl AF et al. *JAMA Network Open.*

eTable 3. Goals of prenatal care as identified by participants

Care goals	Participant Quotes
**Medical Care**	
To optimize health	"[Prenatal visits are] to check up… make sure you're eating good. Picking up weight." *Patient 16, multiparous, postpartum*<br>"A clinic is providing preventive care. They go take care of this problem and so little problems don't become big problems." *Healthcare Worker 1*
To provide reassurance	"They [prenatal visits] be helpful to my mind, knowing that his heartbeat is very good and, you know, like there's nothing wrong within my pregnancy." *Patient 11, multiparous, 23 weeks*<br>"Yeah, I think she was just going because – I mean, she wanted to know that her baby was doing well…" *Healthcare Worker 13*
**Anticipatory Guidance**	
To provide education on pregnancy, birth, the postpartum period, and parenting	"Your first child, your second child, that's when the most critical help that people need is in the first and second child. Critical. You don't know nothing." *Patient 11, multiparous, 30 weeks*<br>"Here's a lady with one child, first pregnancy. This lady down here had four babies. She knows every in and out, but she still can learn. Everybody can learn. Every pregnancy is not the same. Each one is different." *Healthcare Worker 8*
**Psychosocial Support**	
To identify material needs and provide resources	"What we do is to assist in finding out some information or resources that's going to help them to become more stable, because they have a lot of needs. They'll come in here homeless, or going from house to house, dealing with brokenness, dealing with food insecurities… So, it's to our advantage to help them to do what they need to do. We search, research information to be able to help them to do that. *Healthcare Worker 15*<br>"Yes, it's [the clinic] like a supportive place to go if you need the help and stuff like that. They'll help you with diapers, clothes, everything you need. And so since this little human father not around to help, I'm like, I'm happy I found this place." *Patient 2, primiparous, postpartum*<br>"I don't think it's enough to say, oh, sorry your house burned down yesterday, thank you for coming, but not being able to say, here is how we can help you." *Healthcare Worker 5*
To provide social support	"It requires a lot of… I would call them "aunties" of the community in a sense. It requires a lot of love that you may not get from your immediate family, but you are able to get from a service and they treat you like they really love on you. And that's really what's required, and that's how we see the changes. And that is not textbook and that's not system, but it's what's required." *Healthcare Worker 6*

© 2022 Peahl AF et al. *JAMA Network Open.*

eTable 4. Failures of current prenatal care delivery to improve the pregnancy experience and outcomes

Care Failures	Participant Quotes
**Medical Care**	
Prenatal appointments often do not give patients clear medical benefit or reassurance	"I think mothers could better benefit if there was expectation of what happens during each prenatal appointment… this is why you need to come, this is why at the end you come more frequently; you know, things like that." *Healthcare Worker 5*<br><br>"Honestly, I mean, I don't know what's the help about. It ain't no help. It's just to help me let me know that it's getting further and further." *Patient 11, multiparous, 23 weeks*<br>"Because one client told me 'I was pregnant before, I don't have to go every time'. And they don't be doing anything anyway…. I think that she felt that her experience wasn't worth it." *Healthcare Worker 8*
Prenatal visits are low-value for many patients:	"It was like - she's just going to check the heartbeat and it's going to be like 10 minutes. No, I'm not going to waste my gas. Back then, I was surviving so, no, I don't want to waste my gas just to go out there for 10 minutes." *Patient 6, Multiparous, postpartum*<br><br>"I come in the morning. I end up leaving before I had to be at work, because I had to be at work at five in the afternoon. I leave here about three, had to hurry up and rush to get home for work." *Patient 16, multiparous, 20 weeks*
**Anticipatory Guidance**	
Patients have inadequate reliable, easily accessible information	"My doctor's office doesn't really have much, so I joined a social media group, a breastfeeding group." *Patient 1, nulliparous, 25 weeks*
Health care workers lack time and educational resources to share with patients	"I have had a couple clients ask about parenting classes and things like that, and I don't really have a lot of resources to give them." *Healthcare Worker 2*
Patients are not comfortable asking questions	"When you ask a lot of questions and then it looks like you don't know what you're talking about… you don't want to look like you don't know about your own body." *Patient 24, Multiparous, 20 weeks*<br><br>"I think, sometimes, the dynamic between the provider and the patient, they don't feel comfortable asking questions. And I also think that many times if the patient expresses things, it's not explained, or it's blown off or it's "don't worry about it". I don't think enough compassion is given and care with addressing concerns." *Healthcare Worker 11*

© 2022 Peahl AF et al. *JAMA Network Open.*

Online resources and friends and family are readily available but unreliable	“But in other ways, there still is a lot of misinformation that happens talking to people that you know and care about, and people on the internet. I mean some of things you will find, I mean, it’s just… I mean this is wild. It’s not true.” *Healthcare Worker 5*
**Psychosocial Support**	
**Material resources**	
Screening for resource needs is not sufficient	“Like there are some, that is the point where they're supposed to be asking them, you know, do they need additional things? In a lot of places, they probably don’t.” *Healthcare Worker 16*
Accessing resources is complex, requires significant assistance	“Nowadays, you got to ask people about their resources. They barely want to give them up. Or you got to dig, go on Google and keep looking, keep looking, keep looking. It will take you all day to find an actual good resource.” *Patient 12, multiparous, 20 weeks*<br>“People don’t know a lot of resources. Even though we assume that they do, or there's a sign on the bus or something. They really don’t. So, you really have to guide them a lot and try to give them information or network for them to find the right people.” *Healthcare Worker 16*<br>“Like, when you get, when the women do get the information, just like make sure it really works out, or it really can help and not just basically give you the run around.” *Patient 8, multiparous, 28 weeks*
Available resources are insufficient	“We did not have a home, and even before we left the home that we were in, I asked them if they had any resources. They gave me what they had, but it wasn’t really any help…” *Patient 17, multiparous, 29 weeks*
**Social support**	
Patients desire greater partner support	“Even like my friend, like with myself, my friend is more supportive than my boyfriend. This is as far as like little things: having a meal cooked…” *Patient 10, multiparous, 26 weeks*
Current prenatal care structure does not integrate psychosocial support	“What I don’t see happening is the consistency of support. You have scheduled appointments for support but just that being able to reach out and have that person there for them and have easy access to whatever they may need in that moment. That’s what’s missing; not for any wrongdoing, just that’s what’s missing.” *Healthcare Worker 9*<br>“I think a lot of women – even though we really give excellent, I think, prenatal care, and we're watching everything carefully and all the things we should be attending to, that I always feel we're still lacking in the psychosocial care.” *Healthcare Worker 16*

© 2022 Peahl AF et al. *JAMA Network Open.*

Overarching Prenatal Care	
**Maternity care clinicians**	
Short appointments, seeing multiple maternity care professionals in pregnancy, and administrative burden preclude strong relationships between patients and maternity care professionals	"I think a lot of times people just don't feel like they know who they are talking to... But I know like many moms have said they don't want to have their baby with a stranger…I know many practices have like rotating providers. But I have heard lots of moms have expressed that's not something that they would want." *Healthcare Worker 5*<br>"It's [the clinic] student-based but I still thought it would be a little bit more… what's the word I'm looking for… not structured because it's a very structured… it's very consistent but it's just not… like it's hard to tell different people personal stuff." *Patient 10, multiparous, 26 weeks*<br>"I think sometimes the clinic is so fast, you know, the turnover, and everything is time, time, time, time is money. And a couple things, maybe sometimes it's just really too fast. You know there's really so much going on in the office and maybe they feel that doctor – or nurse or anyone – will not really take the time to listen because they are so busy." *Healthcare Worker 11*<br>"When I get ready to have the baby, whoever is at work, that's who's going to be delivering my baby. So, when I found that out, now I'm like very upset. Wait, I'm supposed to be building a relationship with a person through this little time that I have… but you have me meeting a different person every appointment." *Patient 18, multiparous, 39 weeks*<br>"They need to strip the administrative blah, blah, blah, out of prenatal care and replace that with informational things… you can make your visits more helpful because you're not under the gun to turn over patients every few minutes…I guess you could answer more questions." *Healthcare Worker 17*<br>"Like my doctor out there in Illinois where I had my first and second child, he was more like conversational versus my doctor with [this child], there was no like relationship, no friendship, no – she just played her role as a doctor and me the patient. So, it wasn't like any conversation or, you know, how are things going." *Patient 6, multiparous, postpartum*
Maternity care professionals do not address patients' non-medical needs	"They can't tell you who ever single thing that might help the pregnancy along or help the family along. And many times, they may not even know kind of what the family or the mother may need or want… Yeah. I don't think it's enough just to have the provider come in and do their thing." *Healthcare Worker 5*
**Structure**	
Medical care, anticipatory guidance, and psychosocial support are poorly integrated	"So, in the hospital, we have more access to other team members that we think could be helpful to this woman. In the community, we're physically not in the same building – other than WIC and us. We're physically not close by. So, that's one barrier. The other is how do you get in touch with somebody. You can leave a message maybe, but maybe you don't get the message. You can send email, maybe they don't get – you know. There's just more logistics." *Healthcare Worker 5*<br>"Like, you know, it's not a lot of resources for a lot of pregnant women unless you like going to different things." *Patient 9, multiparous, 19 weeks*

© 2022 Peahl AF et al. *JAMA Network Open.*

Patients struggle to receive care and balance other obligations	“So, all providers that I've sent my clients to, or that I know my clients go to, don’t see people outside of the hours typically from nine to five. So, if I work a job – this is where equity is an issue – if I work a job that doesn’t allow me sick time, or isn’t flexible... if I work on an assembly line, it's harder for me to pivot and do those middle of the day visits.” *Healthcare Worker 13*<br><br>“When it got to two weeks, it was a little challenging, a little challenging. It was just once you got to every week, that was the struggle. It’s like the doctor this week, oh, man! So, I was still working and at that time, I was a manager so I was still working, like at that time I was seven months.” *Patient 9, multiparous, 19 weeks*<br>“Yeah, the job, the starting date of it was literally the date of the appointment. So, I was like, I’ll just reschedule it... I wasn’t getting prenatal care in the beginning. I wasn’t getting WIC. I was just working.” *Patient 18, nulliparous, 39 weeks*
Prenatal care is one-size-fits-all and not tailored for individuals	“They shouldn’t have to always be just a clinic mode, and this is exactly how we do it, this is our cookie cutter, you know, we are going to make this mode and you cannot go outside of this box.” *Healthcare Worker 11*

© 2022 Peahl AF et al. *JAMA Network Open.*

eTable 5. Ideal future of prenatal care to improve the pregnancy experience and outcomes

Ideal Future of Prenatal Care	Participant Quotes
**Medical Care**	
Patients should enter pregnancy healthy	"And that they don't have any chronic illnesses that goes along with it, even where you would get diabetes and hypertension caused by the pregnancy, even if they were in perfect health prior to, you want them to have smooth sailing." *Healthcare Worker 4*
Intensity of medical care in pregnancy should be based on risk factors	"If it's not high risk, it shouldn't be treated as high risk. Even the settings of clinics and hospitals is just kind of like counterproductive, in my opinion. So just different setting altogether." *Healthcare Worker 7*
Mental health should be integrated in pregnancy care	"And also, just like having mental health be like a comprehensive part of all medical care so it's like automatically assumed that during pregnancy, that your mental health would be thought of along the way." *Healthcare Worker 2*
**Anticipatory guidance**	
Pregnancy information should be comprehensive, clear, and integrated into prenatal care for all stages of pregnancy.	"[I wish] That you were given these classes to make yourself better and counseling for mental health and being able to actually take care of the actual baby once it arrives." *Patient 14, multiparous, 30 weeks*<br>"I wonder if the education component could be in a more user-friendly format." *Healthcare Worker 3*<br>"It could be trimester-specific; during this trimester, this is what your body is going through. This is what is happening." *Healthcare Worker 18*
Patient should have safe spaces to ask questions and gain information.	"You've got a suicide hotline, why can't you have a pregnancy hotline and like that… Some people aren't able to say what it is that they need, what it is that they want and answers that they want to get. I can't read your mind, the doctor can't read you mind so if you just feel like you could just go on, maybe [indiscernible] and they can get all the key points of what it is that you are really trying to find out." *Patient 3, multiparous, postpartum*
**Psychosocial Support**	
**Material needs**	
Prenatal care should support patients in meeting their basic needs, including housing, nutrition, and safety	"More support for mom during these nine, 10 months and one year after birth, … that could be housing, that could be transportation, that could be financial support…making sure mom has access to healthy foods, and just kind of lay off the pressures of the world and really to focus on bringing in a healthy baby." *Healthcare Worker 6*<br>"I feel like it should be everybody should have at least anything, just a safe haven of peace that they can be in when they are pregnant." *Patient 3, multiparous, postpartum*
Prenatal care should support patients in obtaining health care coverage	"But, for the most part, that not have to worry about the insurance, that the location of the clinic is nearby and transportation… was available for someone to pick them up without having any cost to them." *Healthcare Worker 4*

© 2022 Peahl AF et al. *JAMA Network Open.*

**Social support**	
Patients should have a supportive community to help them navigate pregnancy	"She needs support from her partner, whether your husband or not, you need support. So, he needs to pick up the pace and help in this, not just financially but emotionally as well." *Healthcare Worker 11*<br>"I hope that she has… social support in general between family and friends and a partner. It's like where they don't need no man but it's still so much easier with a partner that can help you raise the child." *Healthcare Worker 2*
Social support should be integrated into prenatal care	"And then in terms of the prenatal care, I would love to see like group pregnancy settings where you would have like built into your prenatal care, now you have this extra social support because in a group setting, if you have five people, that's five more minds to come up with different questions that maybe you didn't think of." *Healthcare Worker 2*<br>"That's a good question. I think the perfect pregnancy experience would be a time for excitement, a time for support – like rallying the whole village around, I guess." *Healthcare Worker 5*
**Overarching Prenatal Care**	
**Maternity care clinicians**	
Desire for meaningful relationships with maternity care professionals	"I don't care if it's a doctor, midwife, whomever, someone who is really engaged, really cares to get to know her, to actually keep her coming to the doctor. Someone who is not – if she does have a question or is doing something questionable, she's not getting a punitive response; she's getting someone who says, why do you feel like you need to do that?" *Healthcare Worker 7*<br>"So, her just being supportive, asking plenty of questions… not being rushed during your prenatal visit…" *Patient 5, multiparous, postpartum*<br>"I just hope that she, you know, feels comfortable enough with her doctor…I just hope that they are so personable that she can talk to them about anything, like whatever, anything, like any little thing." *Patient 10, multiparous, 26 weeks*
Need for maternity care professionals to genuinely care about the patient	"It's important for the doctor to care because if the doctor don't care about your body, how are you supposed to know what's going on?" *Patient 12, multiparous, 20 weeks*

© 2022 Peahl AF et al. *JAMA Network Open.*

Hope for the maternity care professional to coordinate medical and psychosocial aspects of prenatal care	"They [providers] have to be all things. They have to be non-judgmental. They have to be mindful of what they say before they say it. They have to actually show that they are listening, reflecting back. Actually addressing all concerns; not just some concerns, but all concerns. And if it's something they cannot address, they need to make sure that they're putting their patient with the appropriate person to be able to address it." *Healthcare Worker 7*<br><br>"Like, if they see you struggling or something like that, they should automatically start helping you out. Like you can check the income… hopefully, either doctors or somebody can help you find more research on free stuff and stuff like that." *Patient 2, primiparous, postpartum*<br><br>"I would love to see that doulas are not considered visitors, but complementary to the health of that person and their family... and I would like to see more cross-collaboration when pregnancy becomes medical, that we have cardiology seeing that person. We have nutritionists seeing that person. We have nephrology or whatever the person needs, to ensure that that person remains healthy, not just during pregnancy but after they give birth too. And we've coordinated that care very well." *Healthcare Worker 13*
**Structure**	
Care structure should be tailored to patients' needs and preferences	"What is the level of intensity that you want for your birth journey? Those are things that I would like, ideally, for you to be able to have all of those things so that you can create it and design it in the same way you would want to create and design a wedding." *Healthcare Worker 9*<br><br>"Care should always be personalized and so maybe is that like do we have questionnaires that we talk to women about in the very beginning of the pregnancy to help coordinate their care… I think that moms would feel like they are more involved in their care and maybe would be more likely to come to appointments if they feel like, oh, I have set out this path for myself so I will show up." *Healthcare Worker 2*
Prenatal care should be modified to decrease barriers	*Additional care team members:* "A community rep or something… someone that talks to them first then, start getting into everything that is needed to do. I think that could help like break down some barriers a little bit or – and like help the patient feel a little more comfortable..." *Healthcare Worker 3*<br><br>*Flexible care models:* "Well, if the clinic was open until 10 o'clock at night, that person going to get off by six o'clock. They can just drop you off and come back and get you." *Healthcare Worker 1*<br>"The video, I kind of considered a video sometimes too, instead of going in, like, if I ain't feeling too good. So, that's good." *Patient 16, multiparous, postpartum*<br><br>"I would love to see prenatal health care available in every neighborhood in [the study city]. Why not? People get pregnant everywhere in [the study city], why do they have to come, again, to those central places…. And I would love to see childcare in the clinical space, so a place where I don't have to worry about my children, if I have older children, my children will be incorporated in that visit, if they're old enough." *Healthcare Worker 13*<br>*Co-location of services:* "It's already in my head: a housing program that you make sure that they are stabilized in their housing… once they're in stable housing, then they're attached to other resources like, a one-stop shop. The OB/GYN and the MIHP, which includes the social worker, registered dietitian, then they would need a resource center to make sure that they're going to be cared for, so that's a part of it… that person is going to be able to have a chance." *Healthcare Worker 15*

© 2022 Peahl AF et al. *JAMA Network Open.*
